# Staphylococcal Superantigen-Like Protein 1 and 5 (SSL1 & SSL5) Limit Neutrophil Chemotaxis and Migration through MMP-Inhibition

**DOI:** 10.3390/ijms17071072

**Published:** 2016-07-05

**Authors:** Kirsten J. Koymans, Adinda Bisschop, Mignon M. Vughs, Kok P. M. van Kessel, Carla J. C. de Haas, Jos A. G. van Strijp

**Affiliations:** Department of Medical Microbiology, University Medical Center Utrecht, 3584 CX Utrecht, The Netherlands; A.Bisschop@umcutrecht.nl (A.B.); M.M.Vughs@umcutrecht.nl (M.M.V.); K.Kessel@umcutrecht.nl (K.P.M.v.K.); C.J.C.deHaas@umcutrecht.nl (C.J.C.d.H.); J.vanStrijp@umcutrecht.nl; (J.A.G.v.S.)

**Keywords:** *S. aureus*, immune evasion, matrix metalloproteinase, enzyme-inhibitor, neutrophil, innate immunity, staphylococcal superantigen-like proteins

## Abstract

Matrix metalloproteinases (MMPs) are endopeptidases that degrade components of the extracellular matrix, but also modulate inflammation. During bacterial infections, MMPs are important in the recruitment and migration of inflammatory cells. Besides facilitating cell migration by degrading extracellular matrix components, they potentiate the action of several inflammatory molecules, including cytokines, chemokines, and antimicrobial peptides. *Staphylococcus aureus* secretes an arsenal of immune evasion molecules that interfere with immune cell functioning and hamper proper immune responses. An earlier study identified staphylococcal superantigen-like protein 5 (SSL5) as an MMP9 inhibitor. Since multiple MMPs are involved in neutrophil recruitment, we set up an in-depth search for additional MMP inhibitors by testing a panel of over 70 secreted staphylococcal proteins on the inhibition of the two main neutrophil MMPs: MMP8 (neutrophil collagenase) and MMP9 (neutrophil gelatinase B). We identified SSL1 and SSL5 as potent inhibitors of both neutrophil MMPs and show that they are actually broad range MMP inhibitors. SSL1 and SSL5 prevent MMP-induced cleavage and potentiation of IL-8 and inhibit the migration of neutrophils through collagen. Thus, through MMP-inhibition, SSL1 and SSL5 interfere with neutrophil activation, chemotaxis, and migration, all vital neutrophil functions in bacterial clearance. Studies on MMP-SSL interactions can have therapeutic potential and SSL based derivatives might prove useful in treatment of cancer and destructive inflammatory diseases.

## 1. Introduction

Matrix metalloproteinases (MMPs) constitute a large, structurally related, family of zinc-dependent proteases with in the human system currently up to 23 distinct members described. They are named after their initially described role: the turnover and degradation of extracellular matrix (ECM) components [1]. More recently, also non-matrix substrates have been identified for the MMPs, including chemokines, cytokines, growth factors, and receptors [2]. Since then, it has been recognized that they have a much broader function than breakdown of ECM and also play an important role in inflammation and immunity [2,3]. MMPs are directly implicated in bacterial infection, wound healing, and cancer cell invasiveness and they can have both anti- and pro-inflammatory effects, depending on the MMP, the situation, and the target molecule. Chemokines and cytokines can be broken down or converted to antagonists by MMPs, but in recent years, it has been realized that, during bacterial infections, MMPs are widely involved in immune cell recruitment and have been shown to facilitate cell migration to the site of inflammation through several processes [2,3,4,5]. First, breakdown of ECM components opens up a path that can directly lead to enhanced immune cell migration. Secondly, the MMP-induced release of ECM-bound components can result in additional pro-inflammatory signals with chemotactic properties. Thirdly, MMPs directly affect chemoattractant molecules; chemokines can be effectively potentiated through MMP cleavage, thereby enhancing inflammation and aiding in bacterial clearance.

*Staphylococcus aureus* is a highly successful manipulator of the host immune response and has evolved numerous ways to interfere with proper immune functioning [6]. It does so through the secretion of small immune evasion molecules, that bind to and inhibit distinct parts of the immune system, both innate and adaptive. In the defense against *S. aureus*, there are multiple host cells and proteins of importance, with a key role for neutrophils [7]. In order to successfully limit *S. aureus* infections, neutrophils need to be activated, drawn to the site of infection, and extravasate from the circulation. Thus, the secretion of proteins hindering one or more of these processes is beneficial for staphylococcal survival, and, indeed, *S. aureus* secretes several proteins that interfere in these stages. For example, the chemotaxis inhibitory protein of *S. aureus* (CHIPS) interferes with neutrophil chemotaxis through blocking FPR1 and C5aR and superantigen-like proteins 5 (SSL5) and 11 (SSL11) inhibit neutrophil extravasation by blocking the interaction of PSGL-1 with P-selectin [8,9]. Furthermore, SSL5 is described to block the enzymatic activity of MMP9, one of the two main MMPs secreted by neutrophils, to interfere with leukocyte trafficking [10].

MMP9 is not the only MMP involved in antibacterial defense mechanisms; many MMPs have been shown to directly facilitate neutrophil migration to the site of inflammation. MMP1, 8, 9, 13, and 14 are described to enhance two highly important neutrophil chemo-attractants, CXCL8 (IL-8) and CXCL5 (ENA-78) [11,12,13,14]. Additionally, MMP2 works synergistically with MMP9 in vivo to potentiate the action of CXCL5 to promote neutrophil recruitment to the peritoneal cavity in mice [15]. In vivo studies with MMP8 knock-out mice showed deficient neutrophil influx in these mice through impaired release of LIX, the murine homolog of CXCL5 [11]. Furthermore, many MMPs can release the pro-inflammatory cytokine TNF-α from its membrane-anchored precursor [4]. Moreover, IL-1β, which is produced by neutrophils upon *S. aureus* infections and important for proper host defense [16], can be activated by at least MMP2, 3, and 9 [17]. MMP7 is important in neutrophil transepithelial migration and MMP7 deficient mice have inhibited neutrophil recruitment. MMP9 driven proteolysis of collagen has been shown to result in cleavage of fragments with chemotactic potential that stimulate neutrophil migration [18] and MMP1, 2, 3, 9, and 13 induce chemotaxis of human neutrophils and T cells by releasing cyclophilin B [19]. Furthermore, MMP7 has been described to activate pro-α-defensin [20], an antimicrobial peptide, while the hemopexin-like domain of MMP12 might have direct bactericidal activity [21]. Thus, the whole arsenal of MMPs is crucial in a large number of aspects that together allow for optimal neutrophil function. Thus, interference with a large range of MMPs is beneficial for pathogens in order to inhibit proper neutrophil migration and functioning and thereby enhance bacterial survival.

Therefore, we hypothesized that staphylococci secrete additional proteins targeting MMPs to protect themselves from neutrophil-mediated killing. We set up a systematic search for MMP inhibitors by testing a large set (>70) of secreted staphylococcal proteins on the two main neutrophil MMPs: MMP8 and MMP9. We identified SSL1 and SSL5 as potent neutrophil MMP inhibitors, which is for SSL1 its first function ever described. Moreover, we found that the effects of SSL1 and SSL5 are not limited to neutrophil MMPs, but that the staphylococcal proteins are actually broad range MMP inhibitors, inhibiting the full spectrum of human MMPs. We show that SSL1 and SSL5 prevent the potentiation of the important neutrophil chemokine IL-8 and limit MMP-mediated neutrophil migration through collagen. Thus, this study reveals a new function of a staphylococcal immune evasion protein and adds to our understanding of the biological consequences of MMP inhibition by secreted staphylococcal proteins.

## 2. Results

### 2.1. Identification of Two Staphylococcal Inhibitors of Neutrophil Matrix Metalloproteinases (MMPs)

**Figure 1:**
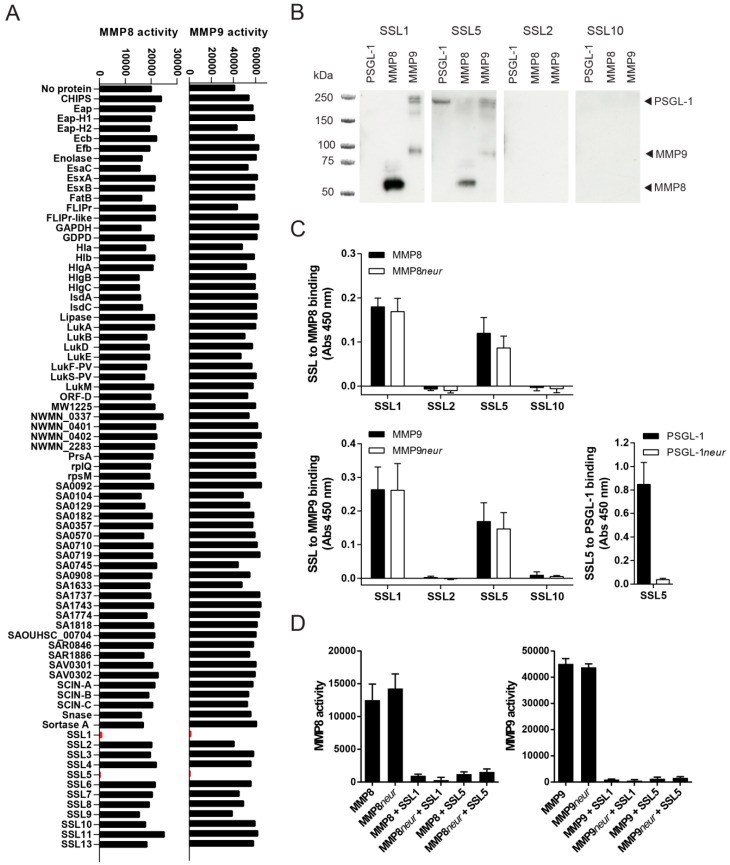
Identification of SSL1 and SSL5 as inhibitors of neutrophil MMPs. (**A**) The effects of 76 secreted staphylococcal proteins (10 µg/mL) on the activity of MMP8 (**left**) and MMP9 (**right**) was assessed by measuring the conversion of a fluorogenic peptide substrate. SSL1 and SSL5 are shown in red. One representative out of three separate experiments is shown; (**B**) SSL to MMP binding was visualized in far Western blot. PSGL-1 (**lane 1**), MMP8 (**lane 2**), MMP9 (**lane 3**), each 0.2 µg/lane, were loaded on gel and subsequently blotted and incubated with 10 µg/mL HIS-tagged SSLs and detected with anti-HIS (anti-X-Press). The expected height of all proteins is indicated on the right. One representative experiment is shown; (**C**) Neuraminidase treatment (*neur*) of 3 µg/mL coated MMP8 (**upper graph**) and MMP9 (**lower graph**) did not alter SSL to MMP binding, as measured by anti-HIS detection in ELISA, whereas it did abrogate SSL5 to PSGL-1 (coated, 3 µg/mL) binding (**right graph**); Data points represent the mean and standard error (SE) from three independent experiments; (**D**) *Neur* treatment of MMP8 (**left graph**) and MMP9 (**right graph**) did not alter the inhibitory activity of 10 µg/mL SSL1 and SSL5 on MMP8/9 activity as measured in the fluorogenic peptide substrate assay. Data points represent the mean fluorescence and SE of three independent experiments.

To determine whether *S. aureus* produces additional MMP inhibitors, we broadly screened for the effects of secreted staphylococcal proteins on the activity of the two most important MMPs secreted by neutrophils: MMP8 and MMP9. Both MMP8 and MMP9 are produced by neutrophils in high amounts, stored in secondary or tertiary granules, and secreted upon neutrophil activation. We incubated activated recombinant MMP8 and MMP9 with 10 µg/mL of 76 different purified staphylococcal proteins and assessed MMP activity by measuring the conversion of a fluorogenic peptide substrate that gains fluorescence upon MMP cleavage of the quenching group. From this screen, we identified two *S. aureus* proteins that inhibit the enzymatic activity of both MMP8 and MMP9: two staphylococcal superantigen-like proteins, SSL1 and SSL5, effectively inhibited the conversion of the peptide substrate, as shown in Figure 1A. None of the other staphylococcal proteins, including the other SSL family members, were capable of inhibiting MMP8 or MMP9. SSL5 was previously described to inhibit MMP9 activity [10], but this screen determined that its activity is not limited towards MMP9. Moreover, for SSL1 this is its first discovered function.

The binding of SSL1 and SSL5 to MMP8 and MMP9 was further confirmed through far Western blotting. PSGL-1, a known ligand for SSL5, MMP8, and MMP9, was run on an SDS-PAGE gel. After the proteins were transferred to blot, they were incubated with SSL1, SSL2, SSL5, and SSL10 and binding of the SSLs was subsequently visualized using anti-HIS detection (Figure 1B). Both SSL1 and SSL5, but not SSL2 and SSL10, can be detected on the lanes run with MMP8 and MMP9, and SSL5 to PSGL-1 binding can also be detected. The specific binding of MMP8 and MMP9 to SSL1 and SSL5 has also been confirmed in ELISA (Figure 1C).

Several of the SSL proteins, including SSL2-6 and SSL11, contain a sialic acid binding motif in their C-terminal β-grasp domain through which they bind glycoproteins. Most previously described functions for SSL5, including PSGL-1 binding, are fully dependent on this sugar-binding motif. Since MMPs can be heavily glycosylated, the interaction between the SSLs and MMPs could also be based on carbohydrate moieties. To examine this, MMP8 and MMP9 were treated with neuraminidase, which removes terminal sialic acid residues, before SSL binding was assessed in ELISA. Neuraminidase treatment did not alter the binding of SSL1 and SSL5 to either MMP8 or MMP9, whereas it did fully abrogate SSL5 to PSGL-1 binding (Figure 1C). Furthermore, neuraminidase treatment did not functionally affect the inhibitory capacity of SSL1 and SSL5 on MMP-mediated fluorogenic peptide conversion (Figure 1D). Thus, the inhibition of MMP8 and MMP9 by SSL1 and SSL5 is not sialic acid dependent, in contrast to the binding of SSL5 to PSGL-1.

**Figure 2:**
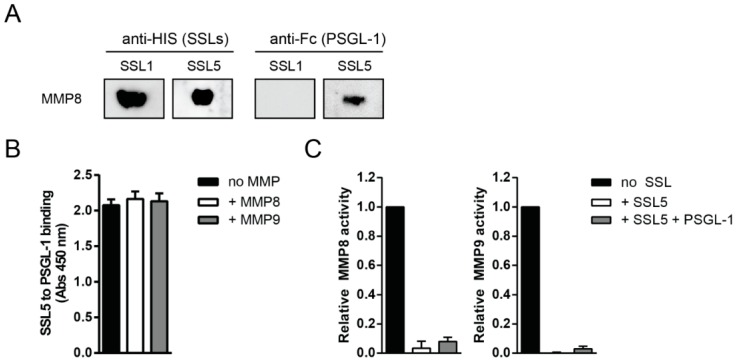
SSL5 binds MMPs and PSGL-1 simultaneously. (**A**) Formation of the triple MMP-SSL5-PSGL-1 complex was examined by far Western blot. MMP8 (0.2 µg/lane) was loaded on a gel that was subsequently blotted and incubated with 10 µg/mL SSL1 or SSL5, after which 2 µg/mL PSGL-1-Fc was added and both SSL and PSGL-1 binding was assessed using anti-HIS (anti-X-Press) and anti-Fc detection, respectively. One representative experiment is shown; (**B**) PSGL-1 (10 µg/mL) binding to SSL5 (3 µg/mL, coated) was not affected by the addition of (10 µg/mL) MMP8 or MMP9 in ELISA. PSGL-1 binding was assessed by anti-Fc detection and data points represent the mean absorption and SE of at least three independent experiments; (**C**) Addition of a 10-fold excess of PSGL-1 (10 µg/mL) to the fluorogenic peptide substrate assay did not affect the SSL5 (1 µg/mL) inhibitory potential on MMP8 (**left**) or MMP9 (**right**). Data points represent the relative mean fluorescence and SE of at least three independent experiments.

As SSL5 can bind both to the MMPs and PSGL-1, we investigated whether SSL5 can also simultaneously bind to these proteins. Therefore, MMP8 was loaded on an SDS-gel in non-denaturing conditions before a far Western blot was performed. The blots were incubated with SSL1 or SSL5 before incubation with PSGL-1, after which both SSL and PSGL-1 binding was determined. We found that both SSL5 and PSGL-1 were bound to MMP8, indicating formation of a triple complex containing MMP-SSL5-PSGL-1 (Figure 2A). SSL1, which does not bind PSGL-1, was used as a control. Furthermore, in ELISA we found that addition of MMP8 or MMP9 does not affect PSGL-1 binding to SSL5 (Figure 2B), again implying that MMP-SSL5 binding does not interfere with subsequent PSGL-1 binding. Lastly, using the functional fluorogenic peptide conversion MMP activity assay, pretreatment of SSL5 with PSGL-1 did not alter the inhibitory potential of SSL5 on MMP8 and MMP9 (Figure 2C). Together, this shows that SSL5 can simultaneously bind PSGL-1 and MMPs in a sialic acid dependent and independent manner, respectively.

### 2.2. Staphylococcal Superantigen-Like Protein 1 and 5 (SSL1 and SSL5) are Broad-Range MMP Inhibitors

**Figure 3:**
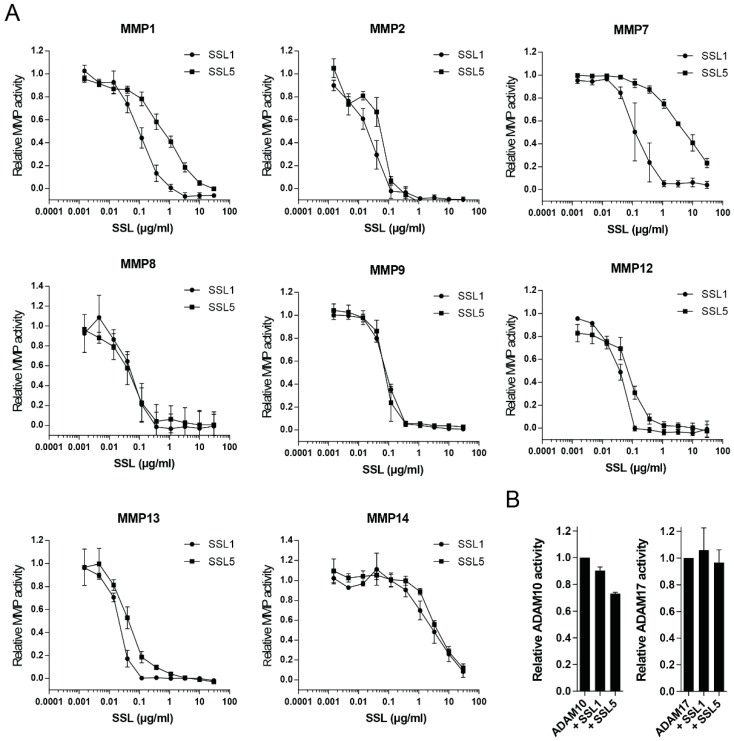
SSL1 and SSL5 are broad-range MMP inhibitors. (**A**) The indicated MMPs were incubated with concentration ranges of SSL1 and SSL5 and MMP activity was measured using the fluorogenic peptide substrate assay. Relative MMP activity was determined based on activity with no SSL present. Data points represent the mean and SE from at least three independent experiments; (**B**) ADAM10 and ADAM17 were treated with a high (30 µg/mL) SSL concentration before activity was assessed in the same assay as in (**A**). Data points represent the mean and SE from at least three independent experiments.

MMP8 and MMP9 are the main MMPs produced by neutrophils, but many other members of the MMP family have been implicated to play a role in neutrophil trafficking and function. More so, the MMP family is highly structurally related and MMP8 and MMP9 are not the closest related family members, both in sequence and structure. This implies that other MMP family members may also be inhibited by SSL1 and SSL5 through a more general mechanism that may be similar to that of endogenous broad-spectrum MMP inhibitors (the TIMPs). Therefore, we tested an array of distinct MMPs that have been suggested to be involved in enhancing neutrophil function. We tested MMP1, 2, 7, 8, 9, 12, 13, and 14 and pre-incubated the activated MMPs with a concentration range of SSL1 and SSL5, before addition of the fluorogenic peptide substrate. All tested MMPs were efficiently inhibited by both SSL1 and SSL5, in a concentration dependent manner, indicating that SSL1 and SSL5 are actually broad range MMP inhibitors (Figure 3A). Activity seems limited to the MMP family however, as the activities of ADAM10 and ADAM17 were not inhibited by a high concentration (30 µg/mL) of either SSL1 or SSL5 (Figure 3B).

### 2.3. The Potentiation of Neutrophil-Attracting Chemokines Is Inhibited by SSL1 and SSL5

**Figure 4:**
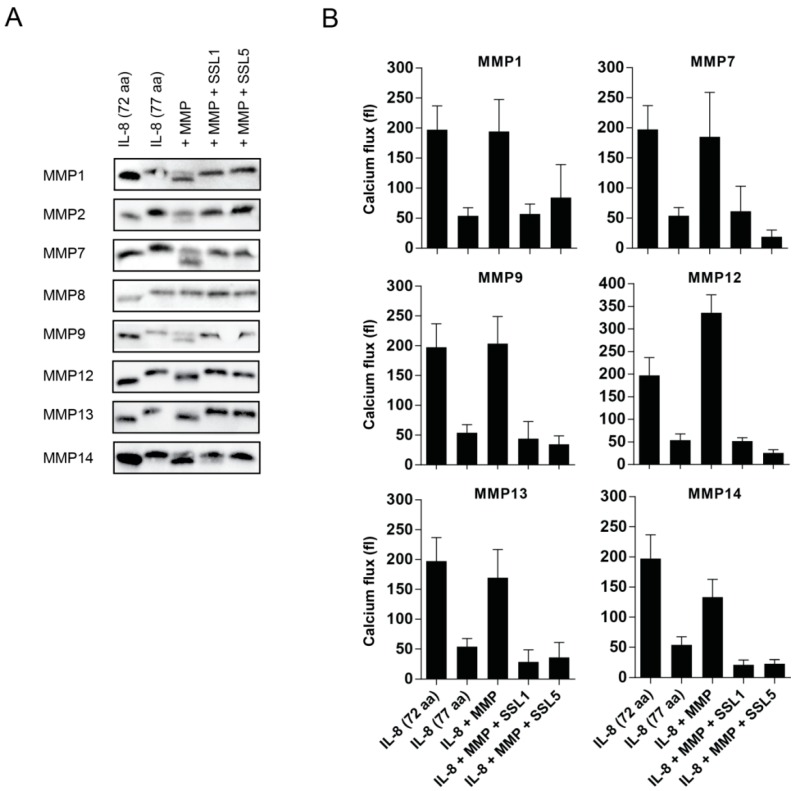
SSL1 and SSL5 interfere with MMP-mediated chemokine potentiation. (**A**) IL-8 72 aa (**lane 1**), IL-8 77 aa (**lane 2**), MMP-treated IL-8 77 aa (**lane 3**), and IL-8 77 aa with MMP pre-incubated with 10 µg/mL SSL1 or SSL5 (**lane 4** and **lane 5**, respectively). All conditions (in each case 0.1 µg/mL IL-8 and 10 µg/mL MMP were used) were incubated overnight and loaded on Tris-Tricine gels. After transfer to blot, IL-8 was visualized with an anti-IL8 antibody. One representative image is shown for all MMPs out of two to three independent experiments per MMP; (**B**) Calcium mobilization in U937-CXCR1 cells was monitored after incubation with different conditions of IL-8. IL-8 (77 aa, 1 × 10^−7^ M) was incubated overnight with the different MMPs (10 µg/mL) with or without 10 µg/mL SSL1 and SSL5 present. The flux was determined by subtracting the mean fluorescence pre stimulus from the mean fluorescence post stimulus. Data represents mean plus SE for at least two to five independent experiments.

MMPs can affect neutrophil function in multiple ways, including the enhancement of leukocyte trafficking through the cleavage of chemokines. The two main chemokine receptors expressed by human neutrophils are CXCR1 and CXCR2 and these receptors play a central role in neutrophil activation, transmigration, and chemotaxis. Their main corresponding ligand, IL-8, requires proteolytic processing to gain full stimulatory capacity and MMPs have shown to be involved herein [3,13]. MMP1, MMP8, MMP9, MMP13, and MMP14 have all been previously described to cleave and potentiate IL-8. During the potentiation, there is loss of only five amino acid residues, but this results in an enhanced IL-8 activity of 3–10 times. Therefore, one of the functional consequences of SSL1 and SSL5 could be to limit the generation of potentiated neutrophil chemokines. To investigate this, we visualized the cleavage of IL-8 by MMPs on Western blot, using an anti-IL8 antibody that recognizes both full length (77 aa) and truncated (72 aa) IL-8. We found that, in our hands, MMP1, MMP2, MMP7, MMP9, MMP12, MMP13, and MMP14 were able to cleave IL-8, and all of these cleavages were fully inhibited by SSL1 and SSL5 (Figure 4A). We could not confirm IL-8 cleavage by MMP8, and, with MMP2, only minor IL-8 cleavage was seen. To continue examining the effects on the inhibition of IL-8 potentiation in a functional assay, we measured IL-8-mediated calcium mobilization through U937 CXCR1-expressing cells for all MMPs that showed clear cleavage on Western blot. After overnight incubation of full length IL-8 with all tested MMPs, an increase in calcium mobilization on U937-CXCR1 cells is seen, that corresponds to the activity of the shorter, more potent, IL-8 (Figure 4B and Figure S1 for a representative example of the calcium flux image). In the presence of both SSL1 and SSL5, this increase is abolished, showing that SSL1 and SSL5 inhibit the MMP-mediated IL-8 enhancement. Thus, SSL1 and SSL5 interfere with neutrophil chemotaxis.

### 2.4. SSL1 and SSL5 Inhibit Neutrophil Migration through Collagen

**Figure 5:**
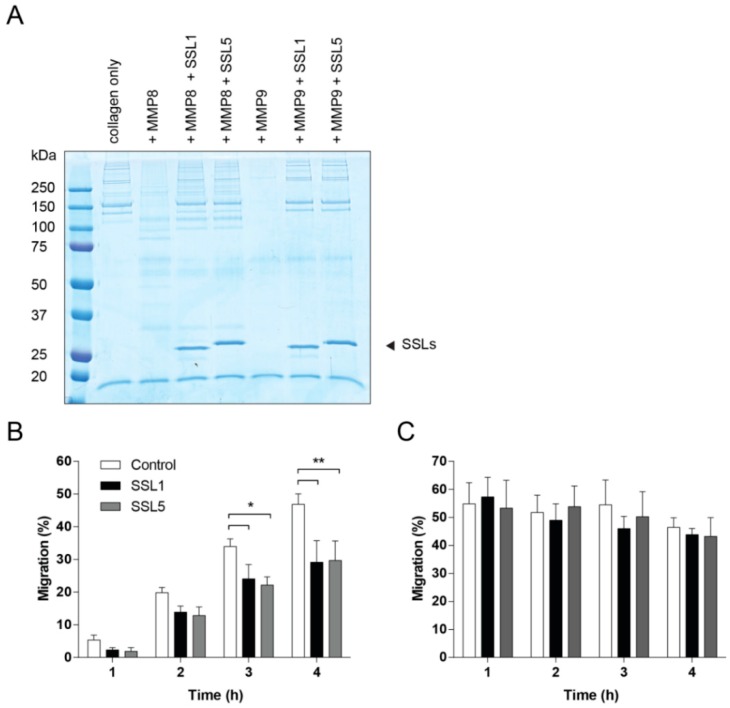
SSL1 and SSL5 limit neutrophil migration through collagen. (**A**) MMP-mediated collagen degradation was visualized using SDS-PAGE. Collagen (0.5 mg/mL) was incubated overnight with MMP8 and MMP9 (10 µg/mL) with and without the SSLs (10 µg/mL). Samples were loaded on gel and visualized using Instant Blue; (**B**,**C**) Migration of neutrophils was assessed in presence (**B**) or absence (**C**) of a collagen gel layer in the upper compartment of a Transwell system. Fluorescently labeled neutrophils, untreated (white bars) or treated with 10 µg/mL SSL1 (black bars) or SSL5 (gray bars), and placed on top of the collagen layer, were allowed to migrate for 1, 2, 3 and 4 h through the gel towards the chemo-attractants fMLP and LTB4 present in the lower compartment. Migration was monitored every hour by measuring the amount of fluorescence present in the lower compartment. Data points represent mean plus SE for at least three independent experiments. * *p* ≤ 0.05, ** *p* ≤ 0.01.

To effectively reach the place of infection, neutrophils need to migrate through the ECM, of which collagen is the major constituent. Several MMPs are capable of breaking down collagen, including the two neutrophil secreted MMPs, MMP8 and MMP9. We first visualized collagen degradation by the neutrophil MMPs and confirmed the inhibition thereof by SSL1 and SSL5 (Figure 5A). Next, we assessed whether SSL1 and SSL5 affect the migration of neutrophils through a collagen matrix. Therefore, a Transwell system was set-up with a collagen gel layer in between chemo-attractant and neutrophils. Neutrophils were stimulated with the peptide and lipid-based chemo-attractants fMLP and LTB4 to prevent inhibition of migration by SSL5 through its interaction with chemokine receptors [22]. Neutrophils were allowed to migrate through the gel for up to 4 h and migration was assessed every hour. Addition of SSL1 and SSL5 showed a decrease of neutrophil migration at all time-points and significant inhibition of neutrophil migration was seen after 3 and 4 h (Figure 5B). When neutrophils were allowed to migrate without the presence of a collagen layer, no inhibition by SSL1 and SSL5 could be detected. Here, maximum migration was already reached after 1 h (Figure 5C). These results show that the SSL1 and SSL5-induced inhibition of migration is collagen-dependent. Thus, SSL1 and SSL5 interfere with neutrophil migration through the ECM.

## 3. Discussion

MMPs are important host factors in fine-tuning immune responses and in the defense against invading pathogens. During many bacterial, including staphylococcal, infections, several MMPs are upregulated in different cell types, to enhance local immune cell trafficking and to aid in bacterial clearance. Exposure to staphylococci induces upregulation of active MMP9 in the spleen [23], and human fibroblasts treated with culture supernatant or whole cell lysates of *S. aureus* show enhanced expression of many MMPs [24]. Several staphylococcal evolutionary conserved components have been shown to directly induce MMP expression, including *S. aureus* peptidoglycan and LTA [25,26,27], and staphylococcal bound plasmin has been shown to activate MMP1 [28]. Here, after an intensive and broad screen, we identified two proteinaceous inhibitors of MMPs, the two staphylococcal superantigen family members SSL1 and SSL5.

MMPs supply direct cues for leukocyte migration and activation. The secretion of SSL1 and SSL5 might help *S. aureus* to avoid MMP-mediated immune activation. We have shown two direct functional implications of MMP inhibition by SSL1 and SSL5: limiting chemokine potentiation and inhibiting neutrophil migration. We found that all MMPs tested, besides MMP8, effectively cleave IL-8, which results in a shorter but enhanced form of IL-8. Besides enhancing chemokine function, MMPs can also destroy signaling molecules or convert them to antagonists, thereby dampening immune responses. These two seemingly opposite effects exemplify the fine-tuning abilities of MMPs. This will affect the spatiotemporal inflammatory conditions in infections and thereby strongly influence the outcome of disease. Interestingly, IL-8, the most important chemokine involved in neutrophil chemotaxis and migration is specifically potentiated by MMPs [2,3]. Thus, for staphylococcal infections, in which neutrophils play a key role in bacterial clearance, inhibiting MMP activation could be greatly beneficial for bacterial survival. The potentiating effects however are not limited to neutrophil chemokines. CCL15 and CCL23 were also shown to be potentiated by MMP-mediated cleavage to promote monocyte recruitment [29]. Furthermore, by breaking down components of the extracellular matrix, besides directly facilitating immune cell motility, the ECM-fragments can be a source of chemotractant potential, also called matrikines [30]. MMP1, MMP2, MMP8, MMP9, and MMP12 have all been implicated in the formation of these fragments with chemotactic potential. The chemotactic fragments have also shown to induce inflammation that in turn leads to more production of MMPs, thereby initiating a circle of inflammation [31]. Thus, by preventing MMP-induced extracellular matrix cleavage, SSL1 and SSL5 inhibit neutrophil migration in several ways. They preserve the physical barrier formed by dense ECM matrices, thereby preventing the formation of an accessible path of migration. Moreover, by inhibiting ECM component cleavage, SSL1 and SSL5 prevent formation of ECM-derived chemotactic peptides. Earlier described chemotaxis inhibitors of *S. aureus* generally inhibit migration by interfering with chemokine-receptor interactions. The migration that we measured through collagen matrices is a special case and highly dependent on MMPs. In our view, this migration in more complex in vitro systems is more close to the in vivo situation, and is, therefore, of high clinical relevance.

MMP8 and MMP9 are produced in high amounts by neutrophils, but many more MMPs have immune potentiating effects. Other cell types that secrete a lot of different MMPs are epithelial and endothelial cells, and macrophages [3]. Epithelial cells, besides producing MMPs, are also capable of producing IL-8 and thus interfering with IL-8 potentiation through broad-spectrum MMP inhibition would be advantageous for *S. aureus* to limit initial danger signals by epithelial cells. Mechanistically, the broad-range MMP inhibition by the SSLs resembles the activity of the endogenous broad-spectrum MMP inhibitors, the TIMPs. All MMPs consist of a minimal domain containing a signal peptide, a pro-domain, and a catalytic domain. Most MMPs contain an additional hinge region followed by a hemopexin-like domain that can be involved in mediating protein–protein interactions and can facilitate binding to the TIMPs [1,32]. MMP7 only consists of the minimal domain and is still effectively inhibited by SSL1 and SSL5, indicating that binding and inhibition is most likely in the minimal catalytic domain. Interestingly, the only pronounced difference in activity between SSL1 and SSL5, SSL1 being approximately 60 times more active than SSL5, was found for MMP7. This could indicate that SSL5 might use an additional region in the MMPs to strengthen its interaction. A few MMPs contain a transmembrane region and are membrane-bound. MMP14, the only membrane-type MMP that we tested, is less efficiently inhibited by SSL1 and SSL5 as compared to the other MMPs. In that sense the inhibitory activity of the SSLs appears to mostly reflect that of TIMP-1, which is also a weak inhibitor of the membrane MMPs [32]. Unlike most TIMPs, the SSL activity seems limited to the MMP family, as ADAM10 and ADAM17 activity was unaffected by SSL1 and SSL5 treatment. Structurally speaking, there are interesting parallels between the TIMP and the SSL families: they both contain an N-terminal OB-fold. This leads us to hypothesize that the SSLs might follow a similar mode of inhibition as the TIMPs. To confirm this hypothesis and also understand why the SSL activity is limited to the MMP family, crystal structures of the SSL1/SSL5 and MMP complexes would have to be solved. Structural information could reveal more on inhibitory specificity, which can be useful in therapeutic setting where you might want to target a specific subgroup of MMPs.

The family of SSL proteins is widely involved in staphylococcal immune evasion with emphasis on direct inhibition of enzymes or immune receptors. Like the MMP family, the SSLs are highly structurally related and sometimes share common interaction partners, as shown for SSL3/SSL4 [33], SSL5/SSL11 [9,34], and now for SSL1/SSL5. There are some common principles of inhibition used by the SSL family, including a shared sugar binding motif in the C-terminal β-grasp domain that is involved in interactions with terminal sialic acid residues. For SSL5, this glycan binding motif and sialic acid binding is essential for its interaction with PSGL-1. Itoh et al. previously described that this motif is also involved in SSL5 to MMP9 binding [10], which is in contrast with our own findings. We found no differences in SSL binding after neuraminidase treatment of MMP8 and MMP9 and sialic acid removal also showed no difference in the functional inhibition of the MMPs by SSL1 and SSL5. SSL1 is not previously described to contain the conserved glycan binding motif, in contrast to SSL2-6, and SSL11 [35], which is in accordance with our data that indicates that these glycans are not involved in formation of the specific inhibitory complexes. Additionally, we found that SSL5 can bind to MMPs and PSGL-1 simultaneously, further indicating a different mode of interaction for the two proteins. This is also reflected by the differences in affinities of SSL5 for MMP9 and PSGL-1: the K_d_ for the SSL5-PSGL-1 interaction was found to be 820 nM [9] as compared to the higher-affinity interaction of SSL5-MMP9 (K_d_ of 1.9 nM) [10]. This is indicative of a glycan–protein interaction for PSGL-1/SSL5 *versus* a protein–protein based interaction for the SSLs/MMPs. It is tempting to speculate that the dual binding of SSL5 to MMPs and PSGL-1 increases its potential to inhibit neutrophil-secreted MMP8 and MMP9. Neutrophils express high levels of PSGL-1 and this could ensure a high local concentration of SSL5 at the cell surface of neutrophils, better-suited to directly inhibit neutrophil MMPs upon secretion. We suggested a similar mechanism for TLR2 inhibition by SSL3. Binding of SSL3 to TLR2 was shown to be independent of glycans [36], however, glycan-dependent binding of SSL3 to the cell surface of neutrophils and monocytes increases its TLR2 inhibitory potential [33,37]. SSL5 is also not the first SSL described to bind multiple proteins simultaneously: SSL7 binds both complement C5 and IgA by using its C-terminal β-grasp domain for C5 binding and its N-terminal OB-fold for IgA binding [38]. Since the β-grasp domain of SSL5 is involved in PSGL-1 binding, it is likely that the SSL OB-fold is involved in the MMP interactions. This strengthens the earlier discussed hypothesis that the SSLs could use a similar mode of inhibition as the OB-fold containing TIMPs.

This paper illustrates and adds to our understanding of the host–pathogen interaction, how widespread the immune evasion strategies of *S. aureus* are, and how important it is for staphylococci to prevent neutrophil activation. The fact that a bacterial pathogen produces two MMP inhibitors underscores the importance of MMPs in bacterial clearance. However, further in vivo studies are required to shed more light on this. A study by Calander et al. revealed a protective role for MMP9 in clearance of *S. aureus* in mice [23] and MMP7 deficient mice showed increased bacterial growth [39]. Controlled MMP expression is necessary for an efficient immune response against invading pathogens, but when this activation becomes excessive, uncontrolled MMP activation can lead to detrimental consequences for host cells and lead to tissue damage and eventually immunopathologies [40]. During these situations with excessive MMP activation, SSL-derived therapeutics could be of interest. This study improves our knowledge of the molecular mechanisms of enzyme–inhibitor interactions that could prove useful in therapeutic settings.

## 4. Materials and Methods

### 4.1. Reagents and Chemicals

NS0-expressed ADAM10, ADAM17, MMP1, 2, 7, 8, 9, 12, 13, and 14 were purchased from R & D Systems (Oxon, United Kingdom). They were diluted to a concentration of 100 µg/mL in assay buffer (50 mM Tris, 10 mM CaCl_2_, 150 mM NaCl, 0.05% Brij-35 (*w*/*v*), pH 7.5, for MMP14 supplemented with 5 µM ZnCl_2_) and stored at −80 °C in aliquots until further use. Recombinant human IL-8 (CXCL8, 72 aa and 77 aa) was purchased from PeproTech. All chemokines were diluted to 100 µg/mL in PBS and stored at −20 °C. Mca-K-P-L-G-L-Dpa-A-R-NH2 Fluorogenic Peptide Substrate IX (R & D Systems) was stored as 2 mM in dimethyl sulfoxide (DMSO) at −80 °C. PSGL-1-Fc was purchased from R & D Systems.

### 4.2. Cloning, Expression and Purification of Recombinant Staphylococcal Proteins

SSL1 and SSL5 were cloned and expressed as previously described [9]. In short, they were cloned into the pRSETB vector (Invitrogen, Carlsbad, CA, USA), containing an N-terminal HIS-tag with an additional X-press epitope, and generated in *E. coli* Rosetta Gami (DE3) plysS. Expression was induced with 1 mM Isopropyl β-d-1-iogalactopyranoside (IPTG). All other used staphylococcal proteins were cloned similarly; some are expressed with a shorter N-terminal HIS-tag, using a slightly modified pRSETB vector [33]. Some proteins were expressed in *E. coli* BL21 (DE3). Proteins were isolated from a HiTrap chelating HP column under either denaturing or native conditions and eluted using an imidazole gradient. Proteins were stored in PBS and purity was confirmed with SDS-PAGE (purity > 95%). SSL1 (SAOUHSC_00383) and SSL5 (SAOUHSC_00390) were cloned from *S. aureus* strain NCTC8325. Other tested *S. aureus* proteins are: CHIPS (NWMN_1877), EAP (SAV1938), EAP-H1 (SA2006), EAP-H2 (SA0841), Ecb (SA1000), Efb (SA1003), Enolase (SA0731), EsaC (SAV0289), EsxA (SAV0282), EsxB (SAV0290), FatB (SA0691), FLIPr (SA1001), FLIPr-like (MW1038), GAPDH (SA0727), GDPD (glycerophosphoryl diester phosphodiesterase; SAOUHSC_00897), Hla (Newbould305 1801), Hlb (PHLC STAAU), HlgA (NWMN_2318), HlgB (HLGB STAAU), HlgC (HLGC STAAU), IsdA (SA0977), IsdC (SA0978), Lipase (MW0297), LukA (NWMN_1928), LukB (NWMN_1927), LukD (SAUSA300_1768), LukE (SAUSA300_1769), LukF-PV (O50604 STAAU), LukS-PV (O50603 STAAU), LukM (Acc No: WP 063651016), ORF-D (MW0205), MW1225, NWMN_0337, NWMN_0401, NWMN_0402, NWMN_2283, PrsA (SAOUHSC_01972), rplQ (SAOUHSC_02484), rpsM (SAOUHSC_02487), SA0092, SA0104, SA0129, SA0182, SA0357, SA0570, SA0710, SA0719, SA0745, SA0908, SA1633, SA1737, SA1743, SA1774, SA1818, SAOUHSC_00704, SAR0846, SAR1886, SAV0301, SAV0302, SCIN-A (SA1754), SCIN-B (SA1004), SCIN-C (SAR1131), Snase (SA0746), Sortase A (SAOUCHSC_02834), SSL2 (SAOUHSC_00384), SSL3 (SAOUHSC_00386), SSL4 (SAOUHSC_00389), SSL6 (SAOUHSC_00391), SSL7 (SAOUHSC_00392), SSL8 (SAOUHSC_00393), SSL9 (SAOUHSC_00394), SSL10 (SAOUHSC_00395), SSL11 (SAOUHSC_00399), and SSL13 (NWMN_1076).

### 4.3. Cells

U937 human pro-monocytic cells were obtained from ATCC (American Type Culture Collection) and grown in RPMI 1640 medium supplemented with 100 U/ml penicillin, 100 µg/mL streptomycin and 10% FCS. For stable expression of human CXCR1 in U937 cells, we used a lentiviral expression system. Therefore, we cloned human CXCR1 cDNA (XM_002581) in a dual promoter lentiviral vector, derived from No. 2025.pCCLsin.PPT.pA.CTE.4x-scrT.eGFP.mCMV .hPGK.NGFR.pre, kindly provided by Luigi Naldini, San Raffaele Scientific Institute, Milan, Italy), as described by Michael L. van de Weijer et al. [41]. This altered lentiviral vector (BIC-PGK-Zeo-T2a-mAmetrine; EF1A) uses the human EF1A promoter to facilitate potent expression in immune cells and expresses the fluorescent protein mAmetrine and selection marker ZeoR. Virus was produced in 24-well plates using standard lentiviral production protocols and the third-generation packaging vectors pMD2G-VSVg, pRSV-REV, and pMDL/RRE. Briefly, 0.25 µg lentiviral vector and 0.25 µg packaging vectors were cotransfected in 293T cells by using 1.5 uL Mirus LT1 tranfection reagent (Sopachem, Ochten, The Netherlands). After 72 h, 100 µL unconcentrated viral supernatant adjusted to 8 µg/mL polybrene was used to infect approximately 50,000 U937 cells by spin infection at 1000× *g* for 90 min at 33 °C. U937-CXCR1 expressing cells were selected by culturing in 400 µg/mL zeocin (Life technologies). Blood from healthy volunteers was collected in heparin tubes and neutrophils were isolated by Ficoll/Histopaque centrifugation, as described previously [9]. Informed consent was obtained from all subjects, in accordance with the Declaration of Helsinki. Approval from the medical ethics committee of the University Medical Center Utrecht was attained (METC-protocol 07-125/C approved on 1 March 2010).

### 4.4. Trypsin Activation of the MMPs

As an alternative for the highly toxic APMA, bovine pancreas trypsin (Sigma-Aldrich, St. Louis, MO, USA) was used for the activation of the MMPs. MMP2 was found to be degraded by trypsin treatment, but showed sufficient auto-activity in our assays. MMPs were activated at a concentration of 100 µg/mL in assay buffer (buffer as described, except for MMP14, the buffer of which consists of 50 mM Tris, 3 mM CaCl_2_, 1 µM ZnCl_2_, pH 8.5) for different times (see Table S1), with a final concentration of 5 µg/mL or 10 µg/mL trypsin. Afterwards, trypsin was inactivated with either or a combination of alpha-1 antitrypsin (final concentration of 100 µg/mL, Sigma-Aldrich), soybean trypsin inhibitor (SBTI, final concentration of 100 µg/mL, Sigma-Aldrich), or 1 mM phenylmethylsulfonyl fluoride (PMSF, Sigma-Aldrich). During all assays, controls for the effect of possible residual trypsin activity were performed and these were excluded. ADAM10 and ADAM17 did not require trypsin activation.

### 4.5. Fluorogenic Peptide MMP Activity Assay

Twenty-five microliters of diluted activated MMP or ADAM (see Table S1) was incubated with 25 µL of the distinct staphylococcal proteins for 30 min at room temperature. Following this, 50 µL of Fluorogenic peptide substrate (20 µM) was added, making a total assay volume of 100 µL. Fluorescence intensity was measured directly over time, with a total of 16 min, in the CLARIOstar microplate reader (BMG Labtech, Ortenberg, Germany) using excitation and emission wavelengths of 320 and 405 nm, respectively. When indicated, MMPs were pretreated with 0.2 U/mL neuraminidase (from *Clostridium perfringens*, Roche, Basel, Switserland) for 1 h at 37 °C. When indicated, a 10-fold excess of PSGL-1 (10 µg/mL) was allowed to bind to SSL5 (1 µg/mL) for 30 minutes at room temperature before the activity assay was performed. MMP activity was assessed by determining the area under the curve after subtracting the area from a blank measurement.

### 4.6. Far Western Blot to Detect MMP-SSL Binding

MMP8, MMP9, and PSGL-1-Fc (0.2 µg/lane) were loaded on 10% SDS-PAGE gels in native conditions (no boiling of the samples, and non-denaturing conditions). Samples were transferred to a PVDF blotting membrane, blocked with 4% skimmed milk in PBS 0.05% Tween-20 (PBS-T), after which the membrane was washed and incubated with 10 µg/mL of HIS-tagged SSL1, SSL2, SSL5, and SSL10. Subsequently, the membrane was washed and incubated with anti-X-Press mAb (recognizing the HIS-tag, 1 µg/mL, Thermo Fisher Scientific, Breda, The Netherlands) and goat anti-mouse HRP (1:10,000, Bio-Rad, Hercules, CA, USA). Blots were developed with ECL Western Blotting Substrate (Thermo Fisher Scientific) and visualized on a LAS 4010 imaging system (GE Healthcare, Hoevelaken, The Netherlands). To assess formation of the MMP8-SSL5-PSGL-1 triple complex, MMP8 was loaded on gel. In this case, blots were first incubated with 10 µg/mL of HIS-tagged SSL1 or SSL5 before washing extensively and incubating the blot with 2 µg/mL of PSGL-1-Fc. PSGL-1 binding was determined by a mouse anti-human IgG Fc peroxidase conjugated antibody (1:5000, Calbiochem, Darmstadt, Germany).

### 4.7. MMP-SSL Binding Enzyme-Linked Immunosorbent Assay (ELISA)

Non-activated MMPs and PSGL-1-Fc were coated overnight at 4 °C in a concentration of 3 µg/mL in 96-well Nunc MaxiSorp ELISA plates in 0.1 M sodium bicarbonate buffer. Blocking was performed with 4% skimmed milk in PBS-T for 1 h at 37 °C, after which HIS-tagged SSLs (10 µg/mL) were added and incubated for 1 h at 37 °C. SSL binding was detected using anti-X-Press mAb (1 µg/mL) and goat anti-mouse HRP (0.1 µg/mL, SBI). Washes were performed 5 times with PBS containing 0.05% Tween in between all steps. Fresh tetramethylbenzidine (TMB) substrate was prepared and the reaction was stopped by the addition of 4 N sulfuric acid before the OD_450_ was measured. When indicated, coated MMPs and PSGL-1 were treated with 0.2 U/mL neuraminidase, for 1 h at 37 °C prior to SSL binding. To determine whether MMP binding to SSL5 affects SSL5/PSGL-1 binding, SSL5 was coated in a concentration of 3 µg/mL, after which 10 µg/mL of MMP8 and MMP9 were allowed to bind for 1 h at 37 °C, before PSGL-1 (10 µg/mL) was added and PSGL-1 binding was assessed by a mouse anti-human IgG Fc peroxidase conjugated antibody (1:10,000).

### 4.8. Western Blot to Visualize IL-8 Cleavage

In total, 10 µg/mL of activated MMP, in some cases pretreated with 10 µg/mL SSL1 or SSL5, was incubated with 0.1 µg/mL of IL-8 (77 aa) overnight in activation buffer supplemented with 0.05% human serum albumin. The 72 aa and 77 aa variant of IL-8 were taken along as controls and treated exactly the same as the MMP samples. Samples (10 µL) were loaded on 16.5% Tris-Tricine gels and run for 2 h at 100 V, after which they were transferred to a blotting membrane using Trans-Blot^®^ Turbo™ Transfer System (Bio-Rad). Blots were blocked with 4% skimmed milk in PBS-T. Subsequently, the membrane was incubated with 10 µg/mL anti-IL8 (R & D Systems, clone 6217.111) and goat anti-mouse HRP (1:10,000, Bio-Rad) for 1 h at 37 °C. In between incubation steps, the blots were extensively washed with PBS-T. Finally, blots were developed with ECL (Thermo Fisher Scientific) and visualized on a LAS 4010 imaging system.

### 4.9. Calcium Mobilization Assay

IL-8 (77 aa) in a concentration of 1 × 10^−7^ M was mixed with a final concentration of 10 µg/mL activated MMP, that was in some cases pretreated with 10 µg/mL SSL1 or SSL5 for 30 min at room temperature. IL-8 (72 aa) was taken along as a control, in the same concentration. The mixes were incubated overnight at 37 °C before a calcium mobilization assay was performed on a flow cytometer (FACSVerse). U937 cells expressing CXCR1 were labelled for 20 min at room temperature with 1 µM of Fluo-3-AM (Life Technologies, Carlsbad, CA, USA). Afterwards, cells were washed once and resuspended in RPMI supplemented with 1% HSA, after which they were diluted to 1 × 10^6^ cells/mL and plated out in 180 µL/well. Twenty microliters of stimuli was added to the cells after 10 s of measurement and the relative calcium flux was determined pre and post stimulus.

### 4.10. Visualization of MMP-Mediated Collagen Degradation

To visualize the degradation of collagen by MMPs we used SDS-PAGE. Collagen Type I (0.5 mg/mL) (from human, Sigma-Aldrich) was incubated overnight with MMP8 and MMP9 (final concentration, 10 µg/mL), in absence of presence of SSL1 and SSL5 (final concentration 10 µg/mL). After overnight incubation, 2 × sample buffer was added and samples were run on a 10% SDS-PAGE gel for 1 h at 200 V. Afterwards, the gel was stained with Instant Blue protein stain (Expedeon, Cambridgeshire, United Kingdom).

### 4.11. Neutrophil Migration Assays

To measure migration of human neutrophils through collagen, we set up a Transwell migration assay. Transwell filters (8.0 µm) (Costar) were filled with 40 µL of collagen solution, containing 1.5 mg/mL collagen (rat tail collagen, type I (BD Biosciences) and 8.6 mM NaOH in PBS, prepared on ice). Filters were put at 37 °C for 90 min to induce collagen gelling. The lower compartment of the Transwell system was filled with 600 µL buffer (HBSS, containing 1% HSA (HBSS/HSA)), or HBSS/HSA containing the chemoattractants fMLP (1 × 10^−8^ M) and LTB4 (1 × 10^−7^ M). Human neutrophils (5 × 10^6^ mL^−1^) were loaded with 4 µM Calcein-AM (Molecular Probes) in HBSS/HSA for 20 min, protected from light. After washing in HBSS/HSA, 100 µL labelled cells (5 × 10^6^ mL^−1^) were added to the upper compartment of the Transwell filters on top of the collagen gel. Next, the Transwell filters were carefully placed in the lower compartment 24 wells. Control migrations were also performed without the addition of collagen. To test inhibition of migration, neutrophils were pre-incubated with 10 µg/mL SSL1 or SSL5 for 30 min at room temperature, before Transwell filters were loaded to the lower 24 well compartments. Migration was allowed for 1, 2, 3, and 4 h at 37 °C. At each time point, the Transwell filters were carefully removed from the lower wells and fluorescence of the migrated neutrophils present in the lower compartment 24 wells was measured in a CytofluorII plate reader. After measurement, the Transwell filters were carefully placed back in the lower compartments to allow further migration in time.

### 4.12. Statistical Analysis

Statistical analysis was performed in Prism (GraphPad Software, La Jolla, CA, USA). The data (for Figure 4) was analyzed with a two-way ANOVA with time as repeated measures, followed by Holm-Sidak’s multiple comparisons test to compare treatments.

## Supplementary Materials

Supplementary materials can be found at http://www.mdpi.com/1422-0067/17/7/1072/s1.
