# Supplementary material for: Staphylococcal Superantigen-Like Protein 1 and 5 (SSL1 & SSL5) Limit Neutrophil Chemotaxis and Migration through MMP-Inhibition
Source: Int J Mol Sci. 2016 Jul 5;17(7):1072. doi: 10.3390/ijms17071072 (PMC4964448; doi:10.3390/ijms17071072)
Supplement: Supplementary file 1 [file ijms-17-01072-s001.pdf]

Supplementary Materials: Staphylococcal Superantigen-Like Protein 1 and 5 (SSL1 & SSL5) Limit Neutrophil Chemotaxis and Migration through MMP-Inhibition

Kirsten J. Koymans, Adinda Bisschop, Mignon M. Vughs, Kok P. M. van Kessel, Carla J. C. de Haas and Jos A. G. van Strijp

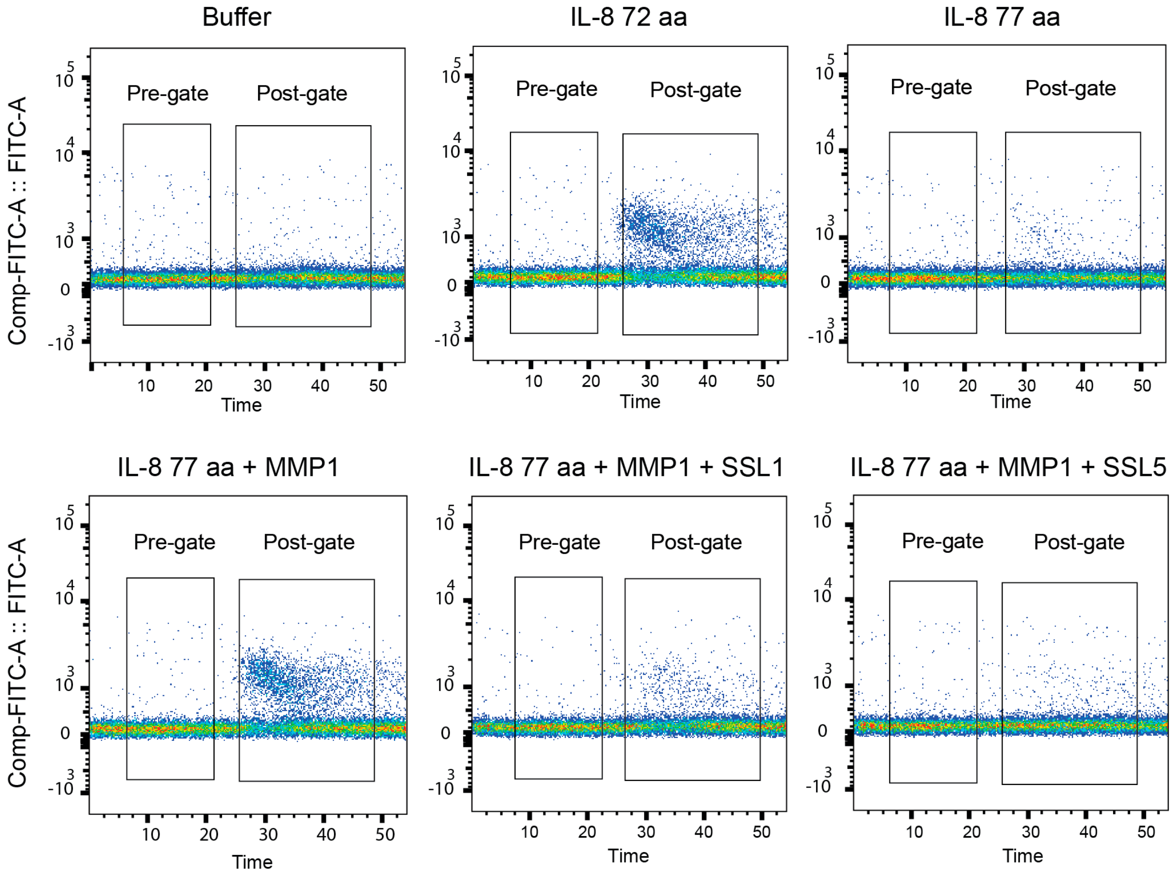

Figure S1. Calcium flux image of a representative experiment with MMP1.

U937 cells stably expressing CXCR1 were stimulated with different variants of IL-8 (all final concentration of 1 × 10^{-7} M): IL-8 72 aa, IL-8 77 aa, IL-8 77 aa treated with MMP (10 µg/mL), and IL-8 77 aa treated with a combination of MMP and SSL (10 µg/mL). The mixes were allowed to incubate overnight before calcium flux was determined. Samples were measured for 50 s (stimulus was added after 10 s) and mean FITC-A fluorescence was determined pre-stimulus (Pre-gate) and post-stimulus (Post-gate). To define the final calcium flux for each sample the mean fluorescence from the Pre-gate was subtracted from the mean fluorescence from the Post-gate.

Table S1. Characteristics and activation of the MMPs and ADAMs.

MMP	Activation	Trypsin Concentration	Concentration in Activity Assay
MMP1 (Interstitial collagenase)	20 min at RT	10 µg/mL	2 µg/mL
MMP2 (Gelatinase A)	2 h at 37 °C	–	4 µg/mL
MMP7 (Matrilysin)	2 h at 37 °C	10 µg/mL	1 µg/mL
MMP8 (Neutrophil collagenase)	3 h at 37 °C	10 µg/mL	2 µg/mL
MMP9 (Gelatinase B)	2 h at 37 °C	10 µg/mL	0.8 µg/mL
MMP12 (Macrophage elastase)	20 min at RT	10 µg/mL	1 µg/mL
MMP13 (Collagenase-3)	30 min at 37 °C	10 µg/mL	0.4 µg/mL
MMP14 (MT1-MMP)	60 min at RT	5 µg/mL	2 µg/mL
ADAM10	–	–	2 µg/mL
ADAM17	–	–	0.8–2 µg/mL
